# Insight into the time-dependent corrosion protection of mild steel by green ionic liquids: a combined electrochemical, surface, and computational study

**DOI:** 10.1039/d4ra08921a

**Published:** 2025-03-17

**Authors:** M. Alharbi, Ruby Aslam, Ajahar Khan, Khalid A. Alamry, Yas Al-Hadeethi, Elena Bekyarova, S. Alqahtani, Mahmoud A. Hussein

**Affiliations:** a University of Jeddah, Collage of Science, Department of Physics Jeddah Saudi Arabia; b School of Civil and Hydraulic Engineering, Chongqing University of Science and Technology Chongqing 401331 China drrubyaslam@gmail.com; c Department of Food and Nutrition, Bionanocomposite Research Center, Kyung Hee University 26 Kyungheedae-ro, Dongdaemun-gu Seoul 02447 South Korea; d Chemistry Department, Faculty of Science, King Abdulaziz University Jeddah 21589 Saudi Arabia mahussein74@yahoo.com maabdo@kau.edu.sa; e Department of Physics, Faculty of Science, King Abdulaziz University Jeddah 21589 Saudi Arabia; f Department of Chemical & Environmental Engineering, Center for Nanoscale Science and Engineering, University of California at Riverside Riverside CA 92521-0403 USA; g Estidama for Project Engineering 7th Floor, Almurjanah Tower Jeddah Saudi Arabia; h Chemistry Department, Faculty of Science, Assiut University Assiut 71516 Egypt

## Abstract

This study reports the development of two green ionic liquids (ILs), namely, choline tyrosinate and choline prolinate, abbreviated as ChoTyr and ChoPro, respectively, as eco-friendly corrosion inhibitors for mild steel (MS) in acidic media. The obtained ILs were characterized by ^1^H-NMR and FT-IR spectroscopy. The anti-corrosive performance of the synthesized ILs was investigated by the weight loss and electrochemical methods (PDP, EIS) in 5% HCl solution at different immersion times of 24, 48, and 72 h at 313 K. The results indicated inhibition efficiencies of 96.9% for ChoTyr, 92.9% for ChoPro in static conditions, 95.5% for ChoTyr, and 91.5% for ChoPro in dynamic conditions after 72 h immersion. The inhibition performance increases as immersion time increases due to the enhanced adsorption of ILs molecules onto the steel surface, forming a protective film. SEM analyses exhibited a smooth surface without corrosion when ILs were present, while untreated steel showed extensive degradation. FT-IR and UV-vis spectroscopic analyses confirmed ILs adsorption. At the same time, theoretical calculations employed using density functional theory corroborated the experimental observations, suggesting stronger adsorption and a higher inhibitory potential of ChoTyr *versus* ChoPro. These findings proved ChoTyr and ChoPro ILs to be sustainable corrosion inhibitors, providing valuable implications for industry applications in acid pickling and cleaning areas.

## Abstract 2

Insight into the time-dependent corrosion protection of mild steel by green ionic liquids namely, choline tyrosinate and choline prolinate, respectively, as eco-friendly corrosion inhibitors for mild steel (MS) in acidic media.

## Graphical abstract

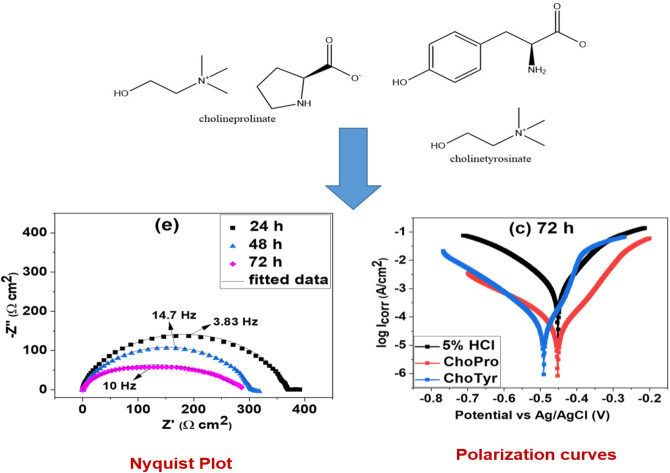

## 1. Introduction

Corrosion inhibitors have received significant attention as potential means to reduce metal dissolution by forming a protective layer on the steel surface, thereby mitigating issues like material loss, expensive repairs, and safety.^1,2^ Recently, ionic liquids (ILs) have gained significant attention as promising green corrosion inhibitors with high thermal stability, tunable molecular structure, and environmental friendliness.^3–6^ Compared with traditional inhibitors, ILs have high efficiency even at low concentrations with low toxicity. El-Nagar *et al.*^7^ examined the corrosion inhibition activity of the imidazolium-based ILs on carbon steel in HCl solution and found that the corrosion rate decreased significantly from 5.95 (μg cm^−2^ min^−1^) (blank) to 0.66, 0.56, and 0.44 (μg cm^−2^ min^−1^) in the presence of 100 ppm of studied ILs at 293 K. Selim *et al.*^8,9^ explored the corrosion inhibiting performance of ionic liquids in inhibiting carbon steel corrosion in 1.0 M HCl. These ionic liquids acted as mixed inhibitors, exerting a maximum corrosion inhibition efficiency of more than 95%. According to another report, the 1-butyl-3-methylimidazolium trifluoromethyl sulfonate([BMIm]TfO) based IL at 800 ppm concentration exhibited 75% efficiency against carbon steel corrosion in the 3.5% NaCl solution.^10^ Lignin-based ILs were used to prevent corrosion of MS in 0.01 M NaCl, with the choline syringe being the most effective, reducing the corrosion current density from 1.66 μA cm^−2^ (blank) to 0.066 μA cm^−2^ after 24 h immersion.^11^ The anti-corrosive performance of 1-methyl-1propyl-piperidinium bromide (MPPB) on Al-6061-10 vol% SiC composite in 0.05 M HCl showed increased %IE with higher MPPB concentration but decreased with rising temperature.^12^ Moreover, Aslam *et al.* have previously reported the corrosion inhibition performances of a few green ILs like proline nitrate,^13^ l-alanine methyl ester nitrate,^14^ ILs derived from amino acid-ester salts,^15^ glycine and glutamic acid-based ILs,^16^ and choline/amino acids based ILs^17,18^ under static conditions and for immersion time of up to 6 h. Although previous studies provided valuable insights into the short-term performance of choline/amino acid-based ILs as corrosion inhibitors, a comprehensive evaluation of their long-term effectiveness under static and dynamic conditions was lacking. This study aims to investigate their corrosion inhibition performance over extended periods and in dynamic environments for a more realistic assessment.

Amino acids, as the building blocks of proteins, have great potential in synthesising ILs owing to their natural biodegradability and bioactivity. They are low-cost, commercially available at high purity, and may be cations or anions contributing to IL properties.^19^ The nature of amino acid, cation–anion and length of alkyl-chain decides the physicochemical and biological properties of ILs. Through molecular engineering, it is possible to tailor the structure of ILs to yield characteristics that minimize toxicity and improve environmental compatibility.^20^ Choline is an essential micronutrient critical for lipid metabolism and transport. Biodegradability, biocompatibility, and cytotoxicity are enhanced in cholinium-based ILs, which promote biocompatibility and biodegradability.^21^ Choline–amino acid ionic liquids are a combination of choline's low toxicity and biodegradability with the versatile properties of amino acids so that they can result in environmentally sustainable materials with promising applications in many fields.^22–24^

The study reports the synthesis of two green ILs derived from choline hydroxide and l-tyrosine and l-proline amino acids, abbreviated as ChoTyr and ChoPro, respectively. Further, the molecular structures of ILs were confirmed using proton nuclear magnetic resonance (^1^H-NMR) spectroscopy and Fourier transform infrared (FT-IR) spectroscopy. The corrosion inhibition performance of these ILs in 5% HCl solution for MS under static and dynamic conditions up to 72 h was studied at 313 K. Various analyses, such as weight loss, electrochemical techniques, and surface analysis, were performed to investigate the inhibition efficiency and elucidate the adsorption mechanism. Theoretical studies using density functional theory (DFT) were also adopted to link molecular properties of ILs to their inhibition performances. Yazdani *et al.*^21^ studied the toxicity and biodegradability of ten choline–amino acid ionic liquids (ILs), reporting low toxicity (EC_50_ : 160–1120 mg L^−1^) and high microbial biocompatibility with minimal impact on Gram-positive and Gram-negative bacteria. The reported ILs exhibited over 60% biodegradability within 28 days, confirming their environmental sustainability. Based on the literature, the ILs under investigation can be considered biodegradable and non-toxic. Therefore, this work aims to demonstrate that ChoTyr and ChoPro ILs are promising green corrosion inhibitors that can find potential industrial applications and contribute to sustainable corrosion management strategies.

## 2. Experimental section

### 2.1 Chemicals and materials

Choline hydroxide (46 wt%), l-proline (≥99%), and l-tyrosine (≥98%) were procured from Sigma-Aldrich, while acetone (>99.0%) and ethanol (>99.9%) were obtained from Merck. All the chemicals were used as received, and the solutions were prepared using double-distilled water. The composition of MS (A1020) under investigation was determined using an optical emission spectrometer (Shimadzu PDA-7000) and reported as follows: (wt%), C (0.28%), Si (0.22%), Mn (0.73%), P (0.015%), S (0.006%), N (0.007%), and remaining Fe. The MS specimens of 3 cm × 2 cm × 1 cm were used for weight loss, UV-vis and FT-IR analysis. Before use, the samples were prepared with SiC (grit size #100 to #3000) papers to give a homogenous surface. After polishing, the specimens were degreased in acetone, rinsed with distilled water, and thoroughly dried. For corrosion studies, a 37% commercial HCl solution was diluted with distilled water to prepare a 5% HCl solution. The inhibitors were applied in the prepared solution at a concentration of 3 × 10^−3^ M (determined based on our previous study^34^).

### 2.2 Synthesis of the materials

Synthesis of both types of ILs involved the coupling of choline to amino acid anions, prolinate and tyrosinate, as described in a previously described method.^19^ Firstly, an aqueous solution of 0.06 mol of the respective amino acid was prepared, and a 4 M aqueous solution of choline hydroxide was added dropwise at room temperature, keeping the corresponding amino acid in slight excess (approximately 10 mol%).

**Fig. 1:**
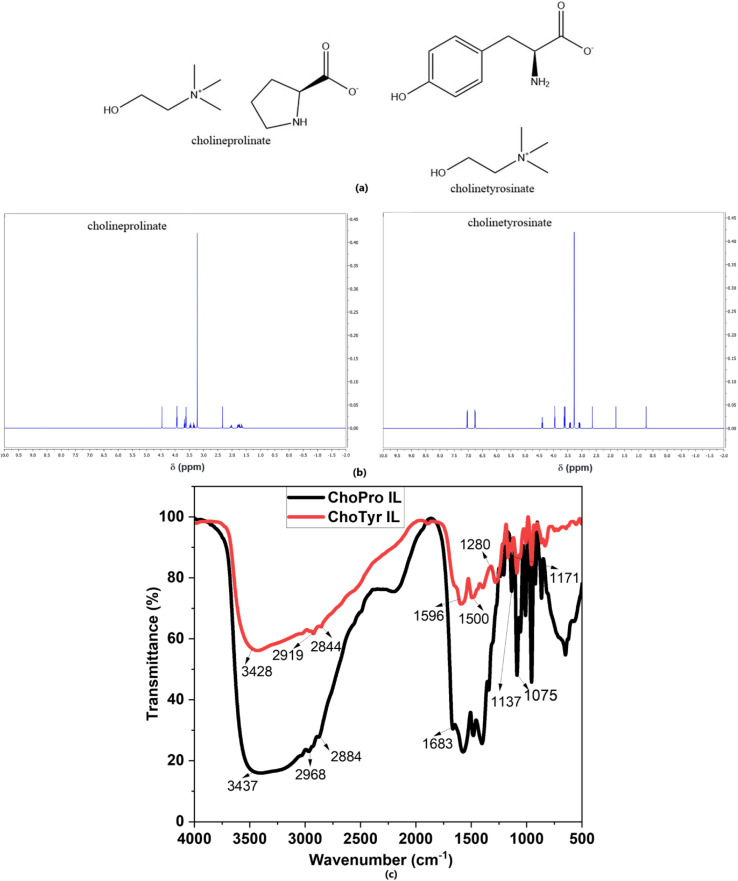
(a) Molecular structures of ILs, (b) ^1^H-NMR spectra, and (c) FT-IR spectra of studied ILs.

The reaction was allowed to proceed overnight at 276 K. After that; water was eliminated under reduced pressure at 323 K using a rotary evaporator. The reaction was mixed well with an acetonitrile-methanol solvent mixture (9 : 1, v/v) to remove leftover amino acids, causing the unreactive amino acids to precipitate. The resulting solution was filtered, and the filtrate was further heated at 323 K to evaporate the solvents. Fig. 1(a–c) shows the molecular structures, ^1^H-NMR and FT-IR spectra of the synthesized ILs, and the obtained data is given in Table 1, along with chemical formulas and molecular weights.

**Table 1:** Characterization data of ILs

ILs	Chemical formula	Molecular mass	FTIR	^1^H NMR
ChoPro	C_10_H_22_N_2_O_3_	218.29	3437 cm^−1^ (OH/NH stretching vibration), 2968, 2884 cm^−1^ (CH stretching vibration), 1683 cm^−1^ (C=O stretching vibration), 1138 cm^−1^ (C–N stretching vibration) and 1075 cm^−1^ (choline C–O–C stretching	(400 MHz, DMSO-*d*_6_, *δ*, ppm): OH– 0.80, CH_2_ & CH_2_ = 3.99 & 3.62, CH_3_ (for 9H) = 3.25, NH = 3.28, 4CH_2_ = 3.50, 1.75, 1.78 & 4.10
ChoTyr	C_14_H_24_N_2_O_4_	284.35	3428 cm^−1^ (OH/NH stretching vibration), 2919, 2844 cm^−1^ (C–H stretching vibration), 1596 cm^−1^ (C=O stretching vibration), 1500 cm^−1^ (C=C aromatic stretching), 1171 cm^−1^ (C–N stretching vibration, choline quaternary amine) and 1280 cm^−1^ (phenolic O–H bending), 1094 cm^−1^ (choline C–O–C stretching)	(400 MHz, DMSO-*d*_6_, *δ*, ppm): 3.45 (s, OH), 3.75 (s, OH), 4.22 (s, NH_2_), 6.62 (dd, 2H), 6.64 (dd, 2H), 2.90 (d, 2H), 2.74 (d, 2H), 2.45 (s, 9H), 2.15 (d, 2H)

### 2.3 Inhibition performance evaluation

#### 2.3.1 Mass loss measurements

The measurements were performed by immersing MS in HCl solution without and with the ILs following the method prescribed in ref. 25. After being cleaned and weighed, the MS specimen (*W*_1_) was immersed in a thermostated water bath at 313 K. The specimens were removed from the solution after 24, 48 and 72 h immersion and cleaned as described above. The specimen was then reweighed (*W*_2_). Using the following formula, the corrosion rates (CR_,_ mm per year) were determined:^26^1
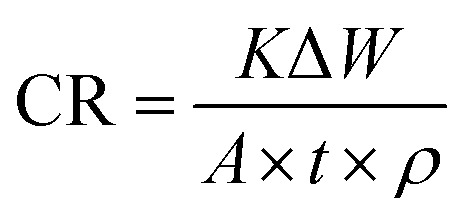
where Δ*W* (Δ*W* = *W*_1_ − *W*_2_) is the weight loss (g), *K* is a constant (8.45 × 10^4^), *A* is the surface area of the MS, and *t* (h) is the end time of each experiment, *ρ* is the density of MS (7.86 g cm^−3^). Eqn (2) was used to calculate the % IE of ILs:^26^2
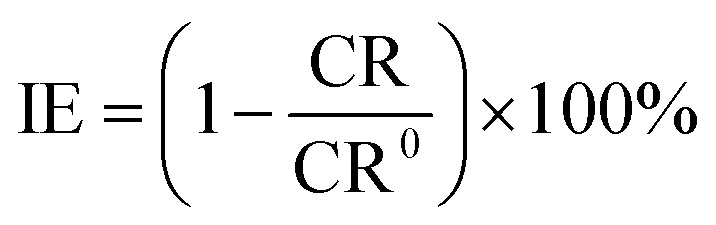
where CR^0^ and CR represent corrosion rates without and with ILs, respectively. Triplicate experiments were performed in each case under the same conditions, and the mean weight loss values were used to calculate the corrosion rates. The mass loss experiment was also conducted under dynamic conditions by placing the beakers on a stirring hot plate at a rotation speed of 410 rpm.

#### 2.3.2 Electrochemical measurements

The tests were conducted using Metrohm Potentiostat/Galvanostat using Model PGSTAT128N. The three electrodes used in this work were KCl (3 M), saturated Ag/AgCl as a reference electrode, a platinum sheet as a counter electrode, and MS as a working electrode. Luggin's capillary connected the reference electrode to minimize ohmic potential drop. Each working electrode, with a 1 cm^2^ exposed area, was embedded into a Teflon holder with an epoxy resin to secure it. A sinusoidal perturbation of 10 mV was used for EIS studies, and the impedance was recorded over a frequency range of 10^−2^ Hz to 10^5^ Hz after 30 min immersion. PDP curves were then measured over a potential of ±250 mV *versus* Ag/AgCl at a scan rate of 1 mV s^−1^. Nova 2.1 software was used to analyze the experimental data. The corrosion current density (*I*_corr_) was calculated by extrapolating the linear Tafel regions of the anodic and cathodic polarization curves to the corrosion potential. All experiments were carried out at 313 K in static solutions. The inhibition performance of MS was calculated using eqn (3) and (4):^27^3
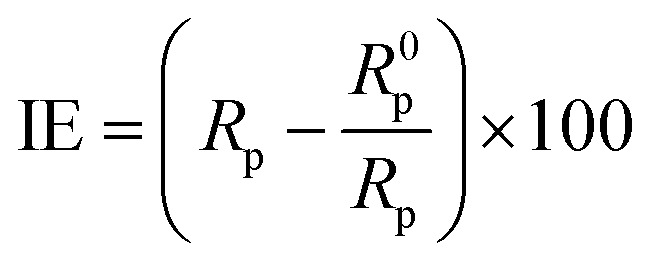
4
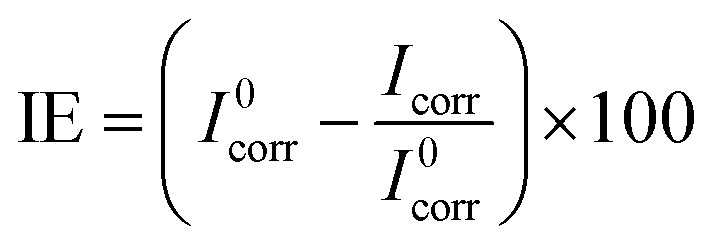
where *R*^0^_p_, *R*_p_, *I*^0^_corr_, and *I*_corr_ are the polarization resistances and corrosion current densities of MS in the absence and presence of varying ILs, respectively.

#### 2.3.3. Spectroscopic analysis: UV-vis and FTIR

Spectroscopic analysis was performed on samples immersed for 24 h in HCl solution without and with added inhibitors. UV-vis spectra were recorded over the 200–700 nm range using a GENESYS 10S analyzer. PerkinElmer spectrometer was used to analyze the FTIR spectra of samples, with spectra recorded in the range of 4000–400 cm^−1^. Like the weight loss measurement section, samples were prepared and immersed in the test solution, after which the film formed on the working electrode would be scrapped and mixed with KBr and pressed into pellets for FT-IR study.

#### 2.3.4 Surface morphological studies

AFM imaging was performed on 1 × 1 mm^2^ MS specimens in non-contact mode using a Bruker Multimode 8 AFM instrument. The surface morphologies of MS immersed in an acidic solution with and without ILs after 24 h immersion were visualized at an acceleration voltage of 15 kV using a scanning electron microscope (JSM-6510L, JEOL). Before the experiments, the samples were prepared as described above and used immediately for microscopic imaging without further treatment.

#### 2.3.5 Theoretical studies

DFT analysis was done in sophisticated theoretical simulations to shed light on the fundamental interactions between ILs and the metal surface and to obtain a thorough knowledge of adsorption behaviour. Molecular electronic properties were assessed using the SPARTAN '18 software^28^ at B3LYP functional^29^ with a 6-311G(2d, p)^30^ basis set. A conductor-like polarizable continuum model (CPCM) was employed to consider the solvent effect.^31^ Several quantum chemical descriptors were calculated using the energy gap (Δ*E*), *E*_HOMO_, and *E*_LUMO_ employing the eqn reported in our previous publications.^17,18^

## 3. Results and discussion

### 3.1 Corrosion inhibition assessment

**Fig. 2:**
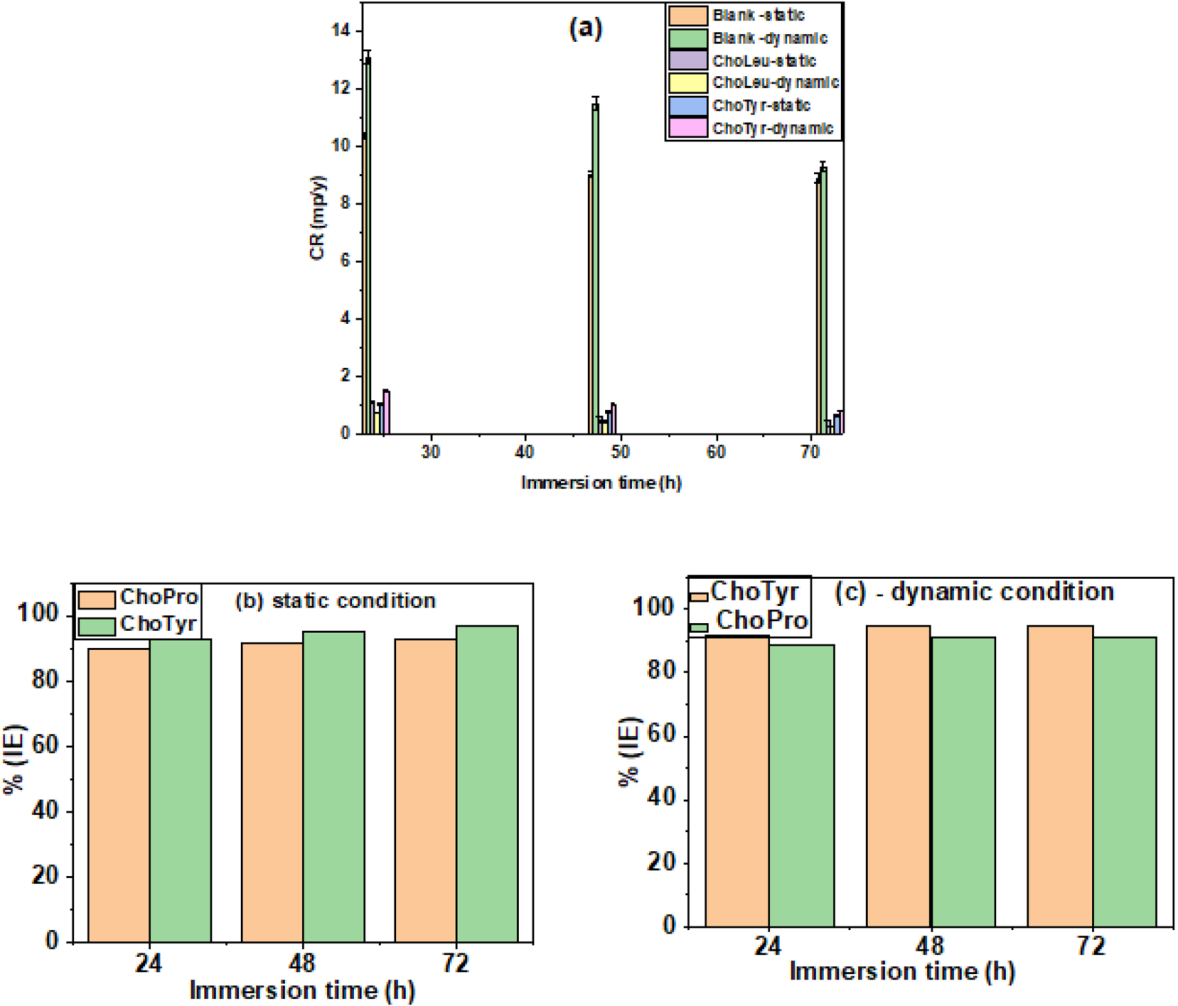
(a) Immersion time *vs.* CR plot in the absence of ILs. (b and c) Immersion time *vs.* % IE plot in the presence of ILs under (b) static and (c) dynamic conditions.

The mass loss tests are used to calculate the % IE by comparing the average weight loss of MS in the absence and the presence of fixed IL concentrations, *i.e.*, 3 × 10^−3^ M. Under static and dynamic conditions and over immersion periods of 24, 48, and 72 h, the corrosion rates and inhibition efficiencies of the studied ILs on MS were compared and evaluated. The results are shown in Fig. 2(a–c).

In the absence of inhibitors, the corrosion rates of MS at 313 K were determined to be 10.3 mm per year, 9.0 mm per year, and 8.89 mm per year under static conditions after 24, 48, and 72 h of immersion, respectively, as shown in Fig. 2(a). Under dynamic conditions without inhibitors, these values increased slightly to 13.0 mm per year, 11.4 mm per year, and 9.2 mm per year due to the influence of flow (Fig. 2(a)). On adding the ILs at a concentration of 3 × 10^−3^ M, the corrosion rates were decreased, and the maximum decreased was obtained as 0.47 mm per year and 0.83 mm per year in case of ChoTyr and ChoPro, respectively at 72 h immersion (Fig. 2(a)) under dynamic conditions. However, in static conditions, a further substantial decline in corrosion rates was exhibited, with the lowest 0.27 mm per year and 0.64 mm per year in the case of ChoTyr and ChoPro after 72 h immersion, as shown in Fig. 2(a). This reduction confirms the ILs' effectiveness in mitigating corrosion. The % IE increased by adding ILs across varying immersion times, as shown in Fig. 2(b) and (c). The order of % IE was static conditions > dynamic conditions in the case of both ILs. The observed trends validate that the ILs act as efficient corrosion inhibitors, forming protective layers on the MS surface that restrict corrosive interactions. Notably, the inhibition efficiency was higher with ChoTyr than with ChoPro IL. This trend suggests a more substantial protective effect of ChoTyr on the MS surface under identical experimental conditions. This may be due to structural differences between studied ILs. Tyrosine contains a phenolic –OH group, an aromatic ring, amine (–NH_2_) and carboxylic (–COOH) groups. The phenolic group enhances its ability to form strong hydrogen bonds and π–π interactions with the steel surface, contributing to better adsorption. Moreover, proline has a non-aromatic pyrrolidine ring and lacks the π-electron system present in tyrosine.

**Fig. 3:**
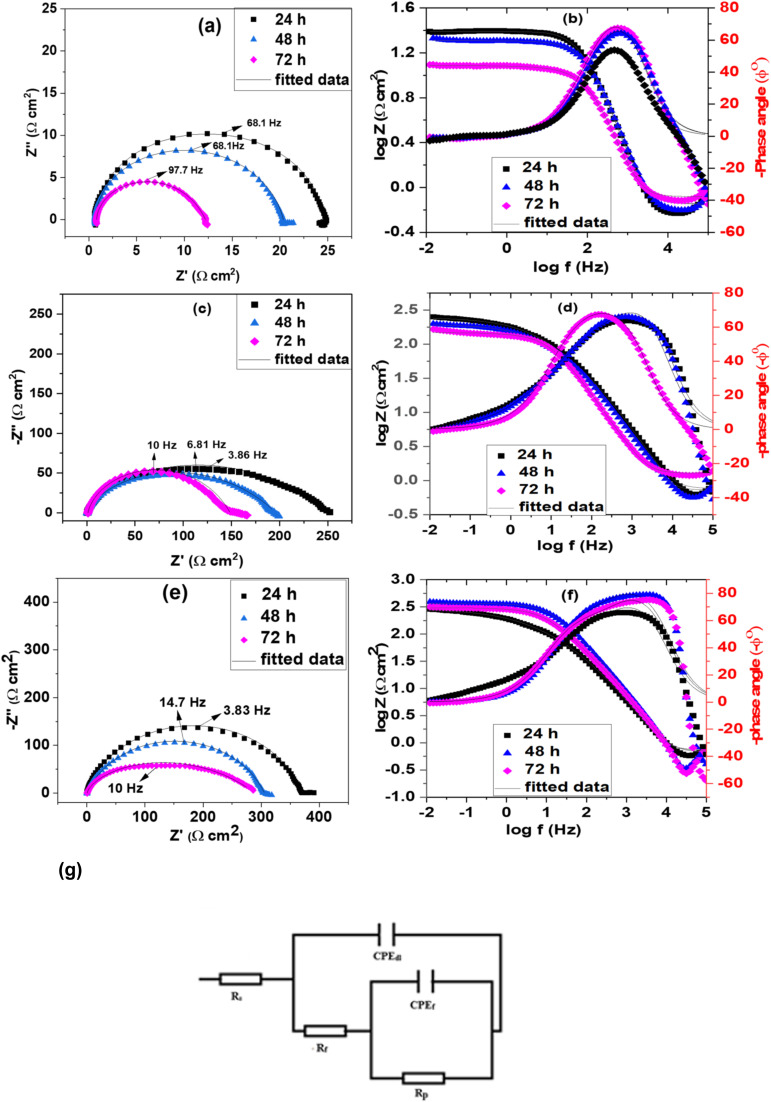
Nyquist (a) blank, (c) ChoPro, (e) ChoTyr and Bode plots (b) blank, (d) ChoPro, (f) ChoTyr of MS in 5% HCl solution at various immersion time at 313 K in the presence of 3 × 10^−3^ M ILs; the open and solid symbols correspond to the fit of the electrical equivalent circuit and the experimental data, respectively (g) equivalent circuit used to fit the impedance data.

EIS data in the form of Nyquist and Bode plots for both uninhibited and ILs inhibited solutions are shown in Fig. 3(a–f). In inhibitor-free solutions at 24, 48 and 72 h, the impedance spectra display a single depressed capacitive loop, indicating that the corrosion process in an acidic solution is primarily driven by a charge-transfer mechanism.^32^ The Bode diagrams demonstrate a single time constant at all frequencies. Nova 2.1.4 software was used to determine the impedance characteristics. The collected EIS data were fitted with the electrical equivalent circuit reported in our previous publication.^33,34^ Among the obtained parameters (Table 2), *R*_s_ stands for solution resistance, *R*_p_ for polarization resistance (sum of *R*_ct_ (charge transfer resistance), *R*_d_ (diffuse layer resistance), and the resistance of any accumulated species such as corrosion products/molecules/ions, *etc.*),^35^ and finally, *R*_f_ represents the film resistance. A constant phase element (CPE) is incorporated into the circuit to achieve a more accurate fit, as the capacitive loops are imperfect.^36^

**Table 2:** Electrochemical impedance parameters for MS in 5% HCl solution without and with ILs at 313 K and varying immersion time

Immersion time	CPE_dl_			CPE_f_									
	*R*_s_ (Ω cm^2^)	*Y*_dl_ (s^n^ Ω^−1^ cm^−2^) ×10^−4^	*n*_d_	*R*_f_ (Ω cm^2^)	*Y*_f_ (s^n^ Ω^−1^ cm^−2^) ×10^−3^	*n*_f_	*R*_p_ (Ω cm^2^)	*R*_total_ = *R*_p_ + *R*_f_	*C*_dl_ (F cm^−2^) ×10^−4^	IE%	*χ* ^2^	slope	−*ϕ*^0^
**5% HCl**													
24 h	0.71	0.87	1	18.25	1	0.90	5.5	23.8	0.87	—	0.004	−0.68	53.8
48 h	0.71	1	1	13.7	2	0.74	5.9	19.6	1	—	0.002	−0.70	64.3
72 h	0.77	2.12	1	6.01	0.72	0.58	6.8	12.8	2.32	—	0.001	−0.73	67.3

**[ChoPro]**													
24 h	0.77	2.39	1	6.05	0.74	0.53	239.8	245.8	0.23	90.3	0.001	−0.70	63.7
48 h	0.72	0.31	1	9.14	0.76	0.54	186.3	195.4	0.31	89.9	0.003	−0.71	66.1
72 h	1.29	0.60	0.99	8.93	0.32	0.66	138.5	147.4	0.60	91.3	0.002	−0.76	67.5

**[ChoTyr]**													
24 h	0.74	0.23	1	66.07	0.86	0.48	281.5	347.5	0.23	93.1	0.003	−0.71	65.9
48 h	0.67	0.184	1	67.88	0.09	0.72	303.7	371.5	0.18	94.7	0.007	−0.82	78.9
72 h	0.70	0.191	1	32.53	0.14	0.71	272.7	305.2	0.191	95.8	0.009	−0.77	75.3

CPE has two elements: a constant (*Y*_0_) and a phase shift indicator (*n*). Eqn (5) can be used to express the impedance of CPE.^38^5
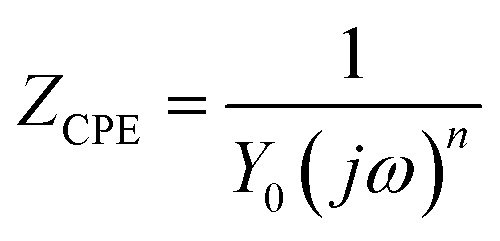
where *j* = √−1 and *ω* is the angular frequency. *Y*_0_ reflects an ideal capacitor when the value of *n* approaches unity. The values of *n* and *Y*_0_ provide information on the film property and the corroding surface. A small value of *Y*_0_ could mean good surface film quality.^37^ The double-layer capacitance (*C*_dl_) values were evaluated using eqn (6).^38^

6 *C*_dl_ = *Y*_0_(*ω*_max_)^*n*−1^

where *ω*_max_ = 2π*f*_max_ and *f*_max_ is the frequency at which the imaginary component of the impedance is maximal.

*Y*_dl_ and *Y*_f_ (Table 2) represent the non-ideal capacitance of the double layer and the protective film, respectively.^39^ Their decrease with immersion time indicates that over time, the IL molecules adsorb more effectively onto the steel surface, forming a denser and more stable film that reduces the ability of ions to penetrate through it. *R*_p_ and *R*_t_ are key indicators of corrosion resistance, with higher values reflecting better inhibition performance.^40^ The increase in *R*_p_ and *R*_t_ indicates that the ILs effectively inhibit the charge transfer reactions involved in corrosion by blocking active sites on the metal surface. *C*_dl_ is inversely proportional to the thickness of the electrical double layer or the dielectric constant of the medium at the metal/electrolyte interface. The adsorption of ILs creates a thicker barrier layer, which reduces *C*_dl_.^41^ This is consistent with the fact that IL forms a protective film that separates the metal from the corrosive environment.

As illustrated in the Bode impedance and phase angle graphs (Fig. 3(b, d and f)), the impedance shifts up, and the phase angle (−*ϕ*^0^) moves closer to −90° in the presence of ILs following the trend ChoTyr > ChoPro. This indicates enhanced corrosion protection as a higher impedance reflects greater surface resistance to corrosion. A phase angle near −90° suggests the formation of a protective film. Notably, this effect becomes stronger with increasing IL concentrations, confirming the concentration-dependent efficiency of the inhibitors.

**Fig. 4:**
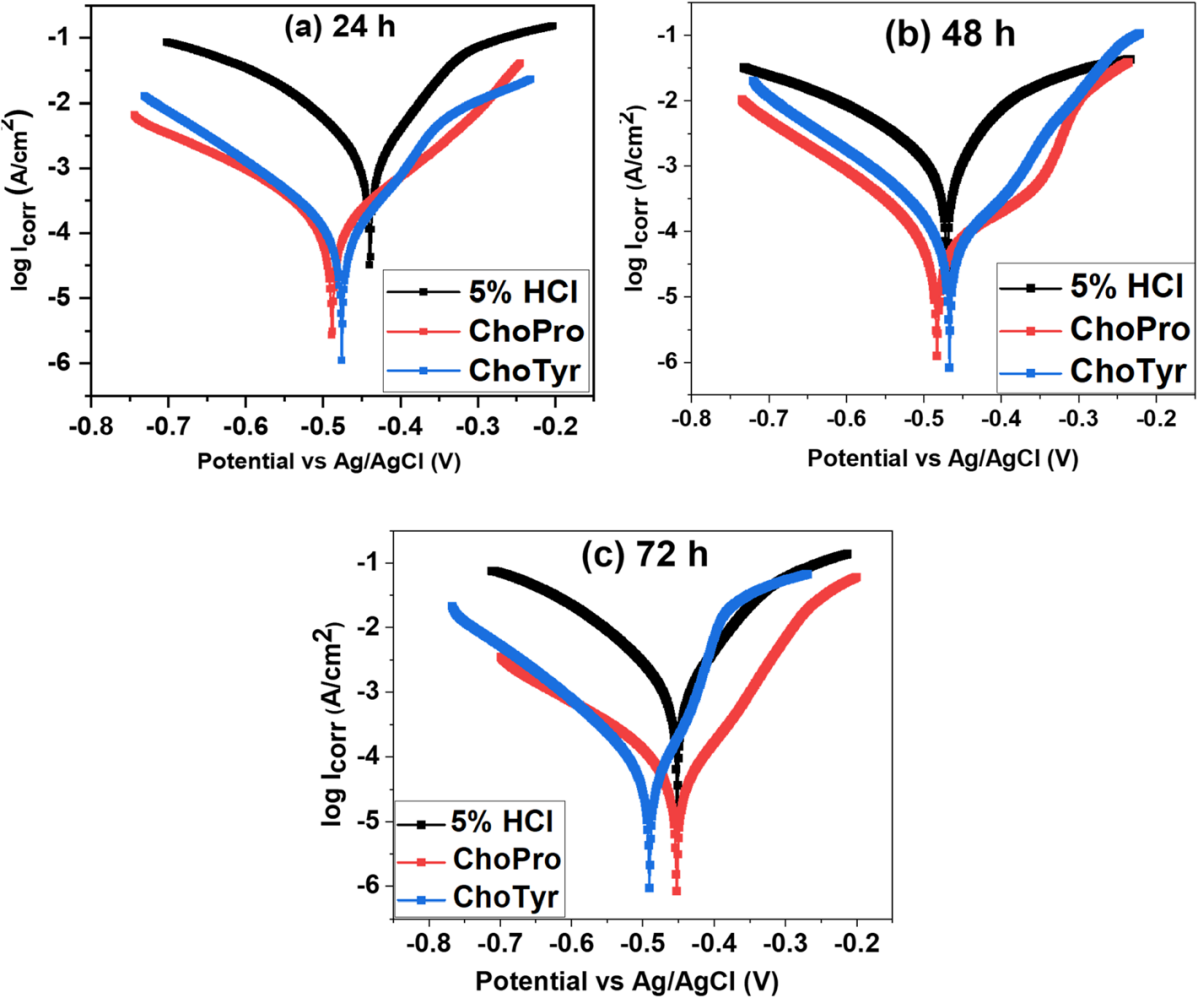
Polarization curves for MS at various immersion times at 313 K in the absence and presence of 3 × 10^−3^ M ILs.

Fig. 4(a–c) shows the potentiodynamic polarization curves, which demonstrate the corrosion behavior of MS in 5% HCl solution without and with ILs at varying immersion times under static conditions. Table 3 shows the obtained polarization data, such as anodic (*β*_a_) and cathodic (*β*_c_) Tafel slopes, the corrosion potential (*E*_corr_) and the current density (*I*_corr_). Adding ILs significantly suppresses both anodic and cathodic reactions compared to the untreated 5% HCl solution.

**Table 3:** Potentiodynamic polarization parameters for MS in 5% HCl solution without and with ChoTyr and ChoPro ILs at various immersion times at 313 K

ILs	*E*_corr_ *vs.* Ag/AgCl (v)	*β*_a_ (V dec^−1^)	−*β*_c_ (V dec^−1^)	*R*_p_ (Ω cm^−1^)	*I*_corr_ (μA cm^−2^)	IE%
**24 h**						
5% HCl	−0.44	13.6	8.7	13.0	1490	—
[ChoPro]	−0.48	9.2	7.2	176.6	149	90.0
[ChoTyr]	−0.47	16.4	8.7	204.7	84.1	94.3

**48 h**						
5% HCl	−0.47	7.7	7.4	21.5	1330	—
[ChoPro]	−0.48	7.5	9.0	359.1	72.1	94.6
[ChoTyr]	−0.46	17.2	9.3	252.8	64.7	95.1

**72 h**						
5% HCl	−0.45	12.8	9.2	19.4	1020	—
[ChoPro]	−0.45	16.2	7.3	414.9	44.0	95.7
[ChoTyr]	−0.49	28.8	9.8	310.6	36.2	96.4

This behaviour indicates that both ILs are mixed-type inhibitors, which can inhibit both anodic (metal dissolution) and cathodic (H_2_ evolution) reactions of the corrosion process. These results align with previous studies.^13,14,42–44^ An analysis of the *I*_corr_ values shows that the MS immersed in untreated 5% HCl solution has a much larger corrosion current density than ILs inhibited solutions for each immersion time studied (24, 48 and 72 h). After 72 h immersion at 313 K, ChoTyr showed the best corrosion inhibition efficiency, 96.4%. The significant decrease in corrosion rate is evidence of ChoTyr serving as an efficient inhibitor of MS corrosion in acid media.

### 3.2. Statistical analysis

Statistical analysis was carried out using two-way ANOVA to compare ILs effect on IE and differences within the techniques (Weight Loss, PDP, and EIS) with the results presented in Table 4. ANOVA was performed using OriginPro 8.0 at a significance level *p* < 0.05. The *p*-value associated with ILs (0.0017) shows there's a significant difference in IE between ChoPro and ChoTyr, suggesting that the type of IL has a significant impact on corrosion inhibition performance of MS. On the other hand, the *p*-value for different techniques (0.21) is greater than 0.05, indicating no significant difference in IEs obtained using different techniques for a specific IL. Therefore, the corrosion inhibition performance is influenced by the IL type rather than measurement technique.

**Table 4:** Statistical analysis of inhibition efficiency based on IL type and technique

	Degree of freedom	Sum of squares	Mean square	*F* Value	*P* Value
Between ILs	1	45.0	45.0	14.9	0.0017
Between WL, EIS and PDP	2	10.5	5.2	1.7	0.210
Model	3	55.5	18.5	6	0.007
Error	14	42.1	3.0	—	—
Total	17	97.6	—	—	—

### 3.3 Spectroscopic insights into metal–inhibitor interactions

**Fig. 5:**
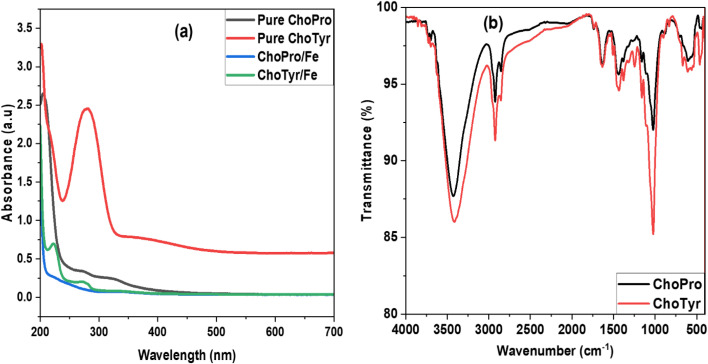
(a) UV-vis spectra of pure ILs and ILs containing solution after immersing MS for 24 h. (b) FT-IR spectra of ILs adsorbed on the metal surface.

UV-vis spectra of ChoTyr and ChoPro ILs before and after immersion of MS for 24 h present noticeably different profiles that suggest adsorption on the metal surface. The spectrum of ChoPro showed an absorbance of 0.34 at 270 nm. This absorption arises from the π–π* electronic transition in the conjugated system of the molecule. Two peaks were detected: 324 nm (absorbance 0.253) and 269 nm (absorbance 0.355) after the adsorption of ChoPro on the metal surface. It indicates the interaction and adsorption of ChoPro onto the MS surface. In another case, when analyzing the spectra of pure ChoTyr, a prominent peak at 280 nm with an absorbance of 2.45 was detected (Fig. 5(a)). This absorption corresponds to the π–π* electronic transition in the benzene ring of tyrosine. The high absorbance value (2.45) suggests that tyrosine has a strong light absorption capability in this region due to its aromatic structure. After adsorption onto the MS surface, the peak shifts slightly from 280 to 274 nm. The appearance of a new peak at 222 nm could be due to a new electronic transition arising from the adsorption of the ChoTyr complex onto the steel surface. This could result from a charge transfer between the metal and the inhibitor molecules, forming a protective layer on the steel surface.^17,42^

FT-IR spectroscopy is a powerful analytical technique widely used in corrosion inhibition studies to gain insights into the chemical interactions between inhibitors and metal surfaces.^45^ The study was done after immersing the MS for 24 h in acidic solution with both studies' ILs (Fig. 5(b)). When comparing the FT-IR spectra of pure ChoPro (Fig. 1(c)) with ChoPro absorbed on the metal surface, all the similar peaks were observed however following shift in the peaks was noticed: N–H stretching vibration and the C=O stretching vibration were observed at 3430 cm^−1^ and 1734 cm^−1^, respectively. Furthermore, the 1424 cm^−1^ and 2928 cm^−1^ peaks reflected N–H bending vibration and N–CH_3_ stretching vibration, respectively. Moreover, the following peak shifts were observed when comparing pure ChoTyr IL (Fig. 1(c)) with the adsorbed IL spectra (Fig. 5(b)) on the metal surface: 3409 cm^−1^ (N–H stretching vibration), 1734 cm^−1^ (C=O stretching vibration), 2928 cm^−1^ (N–CH_3_ stretching vibration), 1427 cm^−1^ (N–H bending vibration). Further peaks were also observed at approximately 1373 cm^−1^ for C–N stretching vibration and at 2846 cm^−1^ for C–H stretching vibration. This shows that when ILs are adsorbed on a metal surface, interactions between the compound molecules and the metal surface usually result in the formation of a thin protective layer, which causes these shifts in the wavenumbers of FT-IR peaks.

### 3.4 Surface morphological studies

**Fig. 6:**
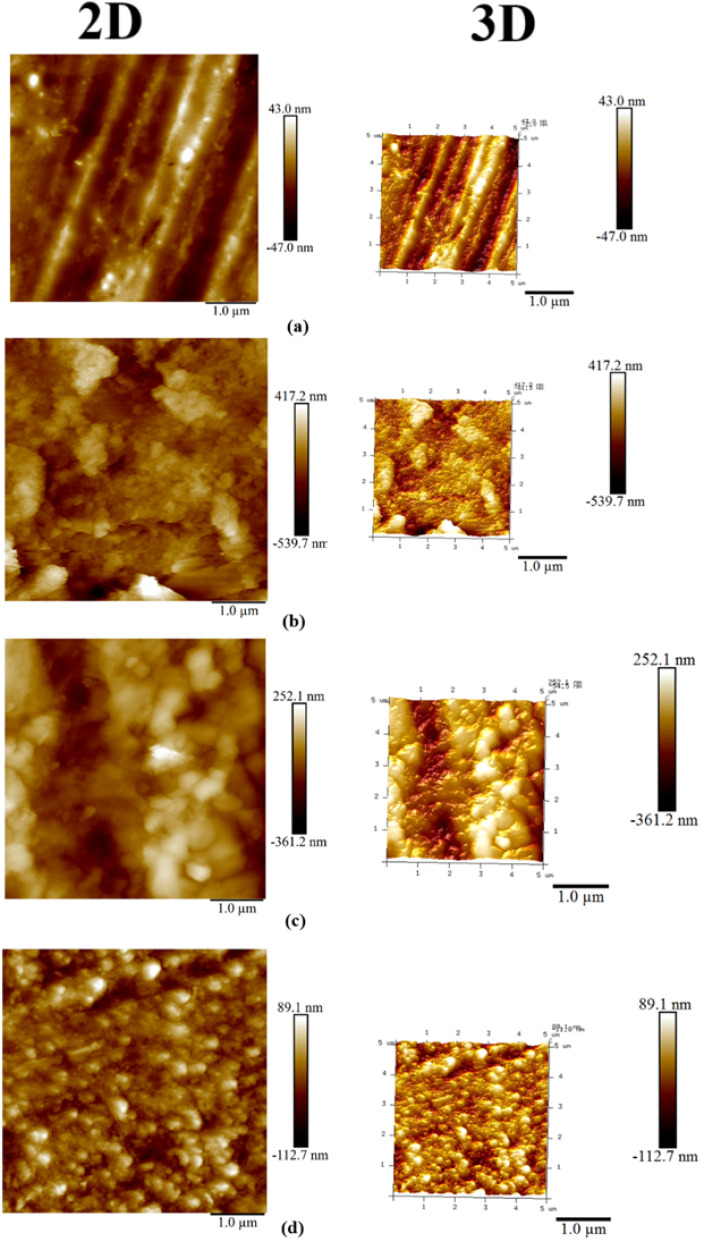
2D and 3D AFM images of MS (a) before immersion in HCl; after immersion in HCl for 24 h (b) without inhibitor (c) with ChoPro (d) ChoTyr.

The Atomic force microscopy (AFM) analysis in the form of 2D and 3D images of the MS coupons reflected the surface topography features under different conditions Fig. 6(a–h). The surface appeared smooth and uniform before immersing the MS in the corrosive medium, as shown in the 2D and 3D AFM images in Fig. 6(a). The surface roughness parameters, such as average roughness (*R*_a_) and the root mean square roughness (*R*_q_), were assessed at 11.4 nm and 14.9 nm, respectively.

The low parameter values suggest that the MS surface in the dry state was in excellent condition, without imperfections on the surface. Upon immersion in a 5% HCl solution without the addition of inhibitors, the surface has been considerably degraded because of the aggressive corrosive attack of the acidic environment, as exhibited in Fig. 6(b). The AFM images of the untreated MS surface imply a highly damaged morphology, increasing the roughness parameters. *R*_a_ and *R*_q_ showed maximum values at 181 and 221 nm, respectively. The observed increase in surface roughness further confirmed the corrosive interaction between MS and acidic solution, developing pits, grooves, and other surface irregularities. On the other hand, ILs showed excellent protective effects, as confirmed by AFM topography examination and illustrated in Fig. 6(c and d). The MS surface treated with ILs was much smoother, and the integrity of the substrate was primarily intact. Notably, the surface roughness values were reduced by both inhibitors, which indicated their efficacy in suppressing corrosion to form protective layers on the MS surface. However, ChoTyr IL (Fig. 6(d)) out-performed ChoPro (Fig. 6(c)). MS surface using ChoPro exhibited *R*_a_ and *R*_q_ values of 71.7 nm and 90.8 nm, respectively. The reduction in the roughness values in the case of ChoTyr IL was much higher, *i.e.*, with values of *R*_a_ as 25.7 nm and *R*_q_ as 33.1 nm, indicating better corrosion inhibition efficiency. The decrease in surface roughness can mainly be ascribed to the effective adsorption and film-forming ability^46^ of ChoTyr IL.

**Fig. 7:**
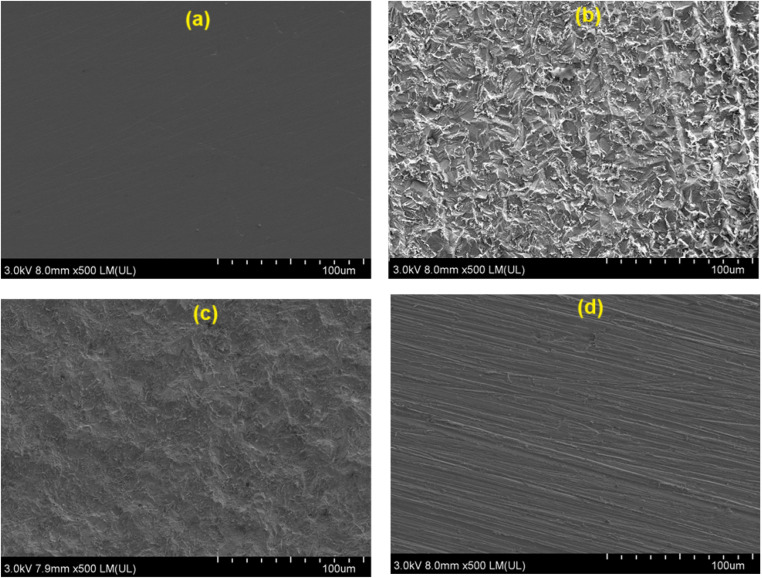
SEM images of MS (a) before immersion in HCl; after immersion in HCl for 24 h (b) without inhibitor (c) with ChoPro (d) ChoTyr.

SEM images of the MS surface before and after immersion in the absence and presence of ILs are presented in Fig. 7(a–d). Fig. 7(a) displays the MS surface after polishing without any imperfections. Fig. 7(b) shows the MS surface subjected to 5% HCl without inhibitors. There is a significant amount of surface damage resulting from the corrosive effect of HCl (Fig. 7(b)). The SEM images Fig. 7(c) and (d) show few defects and a smoother surface when the MS is treated with ILs, suggesting that a protective layer has formed layers that reduce the acid effect.^18^ Among both the ILs, ChoTyr (Fig. 7(c)) showed higher protection than ChoPro (Fig. 7(d)), possibly due to the aromatic phenol group in ChoTyr, which promotes its adsorption on the MS surface under acidic conditions.

### 3.5 Theoretical study

**Fig. 8:**
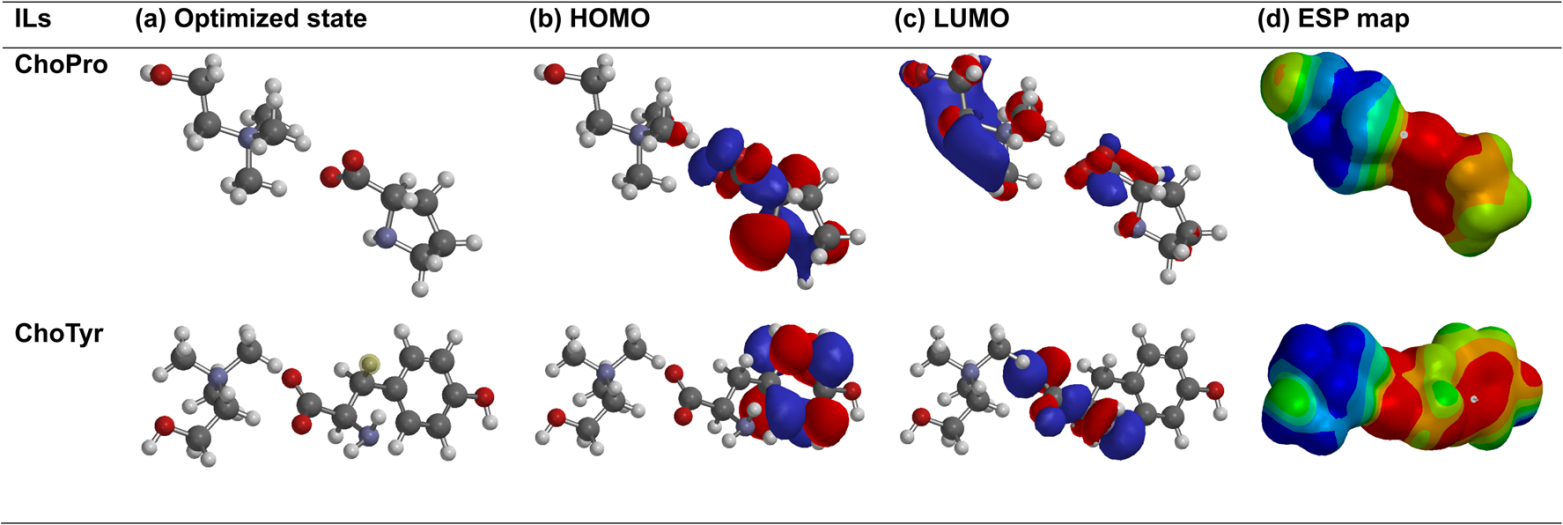
Quantum chemical results obtained by DFT method (a) optimized structures (b) HOMO (c) LUMO (d) electrostatic potential (ESP) of ILs in aqueous phase (grey color ⇒ carbon, white color ⇒ hydrogen, yellow color ⇒ nitrogen, red color ⇒ oxygen).

DFT analysis is one of the most essential aspects for evaluating processes of corrosion inhibitions. As shown in Fig. 8(b–d), the HOMO–LUMO and ESP structures are obtained from the quantum chemical calculations with the geometric structures (Fig. 8(a)). From Fig. 8(b), it can infer that the HOMO is predominantly localized on the aromatic phenol moiety of tyrosine. This is due to the high electron density of the aromatic π-system and the lone pair of electrons on the oxygen atom of the hydroxyl (–OH) group, making these regions the most likely to donate electrons. In the case of ChoPro IL, it is located on the nitrogen atom in the pyrrolidine ring or on the lone pairs of proline carboxyl groups. In both cases, the LUMO is likely localized on the choline group, particularly around the positively charged quaternary ammonium nitrogen, as apparent from Fig. 8(c).

This enables the molecule to accept electron density from the metal during adsorption. Inhibitors with higher *E*_HOMO_ (less negative, destabilized) are likely to have more significant potential for donating electrons to an electron-deficient species, such as a metal surface.^36^ In contrast, a small *E*_LUMO_ (more negative, more stable) indicates the inhibitor's ability toward electron acceptance. The *E*_gap_ is likely to be decreased in ChoTyr due to the presence of the aromatic phenol group with a delocalized π-electron system. A smaller *E*_gap_ signifies better reactivity and adsorption ability. Similar values of both the located HOMO and LUMO orbitals trigger a lower electronegativity (*χ*) (Table 4), meaning that ChoTyr becomes a better electron donor with a lower *E*_HOMO_ and *E*_LUMO_ value owing to the decarboxylated aromatic ring. A greater *E*_gap_ (Table 5) leads to a higher hardness (*η*)^37,47^ and a smaller electrophilicity index (*ω*), indicating a decrease in reactivity, such as that displayed by ChoPro. Lower *χ* and *η* result in a higher Δ*N* (Table 5), meaning ChoTyr transfers more electrons to the metal, forming a stronger protective layer. Fig. 8(d) shows electron surface representations using electrostatic potential mapping (ESP). The red area indicates the high electron density region. In contrast, the blue area indicates areas that have fewer electrons.^48^ The green/yellow region represents neutral regions.

**Table 5:** Calculated theoretical parameters for studied ILs in aqueous media based on a 6-311G(2D, p) basis set at the B3LYP level using SPARTAN '18

	*E*_HOMO_	*E*_LUMO_	Δ*E* (eV)	*I* (eV)	*A* (eV)	χ (eV)	*η* (eV)	*ω*	Δ*N*
ChoPro	−5.77	1.04	6.81	−1.04	5.77	−2.36	3.40	0.82	1.06
ChoTyr	−5.57	−0.28	5.29	0.28	5.57	−2.92	2.64	1.61	1.47

The presence of phenol contributes to forming more pronounced and localized red areas in ChoTyr IL. Besides, the presence of an aromatic group increases its adsorption ability on metal surfaces compared to ChoPro IL. Both molecules have similar blue regions near the choline moiety, indicating comparable electrophilic potential.

The Mulliken charge analysis (Table 6) indicates a significant difference between the electron distribution in ChoTyr and ChoPro, supporting their contrasting efficiencies as corrosion inhibitors. In ChoTyr, the O_3_ atom of phenol has an outstanding negative charge (−0.615) along with the delocalized electron density of an aromatic ring that facilitates an increased donation of electrons and the adsorption on the metal surface. In addition, the carboxyl oxygen atoms (O_1_ and O_2_) and amine nitrogen (N_2_) sites were electron donors with significant negative charges (−0.517, −0.628, −0.560, respectively). The quaternary ammonium nitrogen (N_1_) has a relatively moderate negative (−0.360) charge associated with it, while the neighbouring carbon (C9 = +0.347) has a positive charge, which would favour electron acceptance upon adsorption. Therefore, this equilibrium between nucleophilic and electrophilic character with its delocalized π-electron system gives ChoTyr excellent efficacy in providing a stable covering over the surface of the metal. In ChoPro, the electron density is more localized, with the pyrrolidine nitrogen (N_2_) bearing a notable negative charge (−0.449) and the carboxyl oxygen atoms (O_2_ and O_3_) similarly showing high negative charges (−0.628 and −0.579). However, the lack of an aromatic ring limits the molecule's ability to delocalize charge and interact strongly with the metal surface. The quaternary ammonium nitrogen (N_1_) has a lower negative charge (−0.314) compared to ChoTyr, and the adjacent carbon (C5 = +0.531) is similarly positive, facilitating some degree of electron acceptance. The overall electron distribution in ChoPro indicates a less effective adsorption potential due to weaker electron donation and the absence of aromatic delocalization.

**Table 6:** Mulliken charges for ILs molecules in aqueous media

Atom label	ChoPro	Atom label	ChoTyr
C1	+0.137	C1	−0.119
C2	−0.036	C2	+0.125
C3	−0.174	C3	−0.036
C4	−0.151	C4	−0.112
C5	+0.531	C5	+0.540
C6	+0.046	C6	−0.012
C7	−0.097	C7	−0.112
C8	−0.120	C8	+0.119
C9	−0.008	C9	+0.347
C10	−0.158	C10	−0.144
H16	+0.099	C11	−0.136
H17	+0.094	C12	−0.201
H18	+0.128	C13	−0.147
H19	+0.135	C14	−0.115
H20	+0.296	H21	+0.296
H21	+0.132	H22	+0.092
H22	+0.147	H23	+0.097
H23	+0.134	H24	+0.134
H24	+0.136	H25	+0.140
H25	+0.143	H26	+0.131
H26	+0.135	H27	+0.134
H27	+0.023	H28	+0.139
H28	+0.048	H29	+0.135
H29	+0.061	H30	+0.138
H30	+0.066	H31	+0.134
H31	+0.062	H32	+0.060
H32	+0.065	H33	+0.079
H33	+0.046	H34	+0.077
H34	+0.170	H35	+0.068
H35	+0.136	H36	+0.059
H36	+0.134	H37	+0.078
H37	+0.141	H38	+0.069
N1	−0.314	H39	+0.187
N2	−0.449	H40	+0.191
O1	−0.529	H41	+0.303
O2	−0.628	H42	+0.143
O3	−0.579	H43	+0.139
		H44	+0.140
		N1	−0.360
		N2	−0.560
		O1	−0.517
		O2	−0.628
		O3	−0.615
		O4	−0.479

### 3.6. Inhibition mechanism

The corrosion inhibition mechanism of ChoTyr and ChoPro ILs for MS in 5% HCl is primarily based on their adsorption onto the steel surface, forming a protective barrier. These adsorb through electrostatic interactions and chemical adsorption, aided by tyrosine (specifically, the phenolic and amino groups) and proline (particularly the carboxyl and pyrrolidine groups) functional groups that can form coordinate bonds with the metal surface. This protective film physically insulates the steel surface from corrosive ions, like H^+^ and Cl^−^, inhibiting corrosion.^13,49^ The inhibition behavior of these molecules can be explained based on quantum mechanical parameters. The quantum parameters such as HOMO, LUMO, and electrophilicity index *etc.* indicated that these inhibitors are electron donors and play a significant role in forming a strong protective layer. These ILs inhibit anodic and cathodic corrosion processes and efficiently inhibit corrosion in acidic media.

## 4. Conclusions

This work offers a thorough assessment of the corrosion inhibition efficacy of ChoTyr and ChoPro for mild steel under acidic conditions, utilizing a multidisciplinary methodology that combines experimental methods and computational analysis. Weight loss assessments across different immersion durations validated that both inhibitors significantly reduce corrosion. The inhibitory efficacy remained stable over prolonged durations, signifying the durability of the protective coating on the metal surface. ChoTyr IL demonstrated higher efficiency, *i.e.*, 96.86%, than ChoPro, *i.e.*, 92.73% after 72 h immersion, highlighting its superior long-term performance. Electrochemical techniques further supported the results of the weight loss experiment, suggesting that both inhibitors significantly reduce the corrosion rate of MS, with ChoTyr exhibiting superior efficiency. Quantum chemical studies yielded essential insights into the molecular interactions of the inhibitors with the metal surface. ESP mapping further supports these findings, showing more intense red regions in ChoTyr due to the aromatic phenol group, which enhances its nucleophilic potential and interaction with the metal surface.

Future studies must focus on hybrid formulations containing ILs and other sustainable corrosion inhibitors, as they might yield synergistic effects, hence potentially providing superior corrosion protection. Additionally, this study only focused on two individual ILs for the corrosion protection of MS in acidic media. Expanding this research to include a broader spectrum of ILs, encompassing different structural variations and functional groups, would be beneficial in identifying the most effective candidates for corrosion inhibition. It would also be interesting to test them on different types of metals including alumina, copper, and stainless steel to provide valuable data on their versatility and applicability across industries. Another critical aspect for future exploration is the effect of environmental factors on the stability and efficiency of ILs.

## Data availability

The authors confirm that all data was included in the manuscript.

## Conflicts of interest

There are no conflicts to declare.
